# Pet dogs’ relationships vary rather individually than according to partner’s species

**DOI:** 10.1038/s41598-019-40164-x

**Published:** 2019-03-05

**Authors:** G. Cimarelli, S. Marshall-Pescini, F. Range, Z. Virányi

**Affiliations:** 1 Comparative Cognition, Messerli Research Institute, University of Veterinary Medicine Vienna, Medical University of Vienna, University of Vienna, Vienna, Austria; 2 Domestication Lab, Konrad Lorenz Institute of Ethology, University of Veterinary Medicine Vienna, Vienna, Austria

## Abstract

Most dogs worldwide are free-ranging animals that form relationships mainly with conspecifics, yet research has focused mainly on the dog-human bond, leading to the hypothesis that dogs evolved specific abilities to form a unique relationship with humans. Although widespread, this hypothesis has not, as yet, been tested. Here we compared the relationships pet dogs form with their owner and with other dogs living in the same household. Using a bottom-up approach, we analyzed dogs’ behavior in a test battery with both dog and human partners. Results revealed that pet dogs’ relationships are characterized by three components (i.e. reference, affiliation and stress). A comparison between dogs’ intra- and inter-specific relationships found that overall dogs refer more to their owner, but also that some dogs form stronger affiliative bonds with conspecifics than with their owner. Moreover, we tested how different partners could help dogs cope with a stressful situation. We found that the type of relationship, rather than the partner species, predicts how dogs react to a social threat. Our results suggest that dogs can form relationships of comparable qualities with both humans and other dogs, and that these relationships vary along multiple components across different partners.

## Introduction

Comparable to humans, the domestic dog is one of the most successful mammalian species on Earth^1^. Dogs are distributed across most ecological niches and the global population has been estimated to range from 700 million to 1 billion dogs^2^. In contrast to rodents, whose high abundance is enabled by their cryptic exploitation of human resources and their fast reproduction, the high distribution of dogs has been attributed to their ability to directly interact with humans^3^. It has been suggested that various morphological, hormonal, neurological, behavioral and cognitive skills of dogs have newly emerged specifically to live in human groups and communicate with humans^4,5^ (but see^6^). This human-centered approach to the social evolution of dogs has, however, ignored that most dogs worldwide, although living alongside humans, mainly associate with conspecifics. Even when living as pets, many dogs live within human families in multiple dog-households^7^, not to mention that more than 70% of the entire dog population is constituted by “village” and “feral” dogs^2^. Village dogs inhabit urban or suburban environments and may live either solitarily or in groups of 3 to 27 individuals^7–9^ (see also^10^ for a review). Feral dogs typically live in forested areas with no or minimal human contact, hunting on small prey and scavenging on human refuse^11^.

Living in such variable dog-human groups raises the possibility that rather than having specifically adapted to interact and form “special” bonds with humans (domestication hypotheses)^3,12,13^, dogs might use the same social behavioral repertoire and establish similar bonds with both humans and other dogs.

In order to test this hypothesis, it is necessary to compare the relationships that dogs form with humans and with conspecifics. However, to date such a direct comparison is still missing. Instead, two independent lines of research have investigated either dogs’ inter- or intra-specific relationships. Regarding their relationship with humans, it has been argued that dogs have evolved a novel capacity to form attachment to members of our species, as they use their caregivers for stress alleviation and information seeking similarly to what is observed in human infants^3,13–15^. At the same time, research on dog-dog relationships has focused on other relationship components, such as affiliation and dominance, with results showing that dogs can form strong affiliative bonds with each other that can affect multiple aspects of their life, such as mating preferences^16^, food sharing^17^, cooperation^18^, and so on. These two lines of research focus on different components and different functions of relationships, and the only aspect they share is that both have demonstrated that dogs form differential relationships with both humans^19,20^ and conspecifics^10,21,22^. As such, it is difficult to compare whether the types of relationships established by dogs with humans and conspecifics are in fact different. Hence in the current study we adopted a different approach and aimed at first determining the types of relationships dogs develop with humans and with conspecifics and subsequently investigating whether dog-human relationships are indeed different to dog-dog relationships.

To achieve our aims, we developed a novel test mimicking a series of situations that pet dogs, during their everyday life, may naturally face both with their owners and with other dogs. This so-called “Relationship test” was used to identify various components of pet dogs’ relationships that are not strictly associated with the attachment system^3,13–15^ and to characterize the different types of relationships dogs may establish with human and dog partners. For this aim, we presented dog-dog and dog-owner dyads with situations that involved a chance to explore a novel environment, a separation and reunion phase, and the appearance of a novel, potentially fear-evoking object. An in-depth behavioral analysis of dogs’ behaviors with their conspecific partner and with their owner was carried out, in order to characterize and compare different intra- and inter-specific relationship types. In a second step, to assess the functional role of these different relationships, we presented the same dog-owner and dog-dog dyads with a “Social Threat test” where we compared whether dogs’ reactions to a masked human approaching the dyad in a threatening manner were influenced by the relationship the dogs had with the partner present.

## Results

### Profiling and comparing dog-dog and dog-human relationships

A total of 70 subjects were tested with a dog partner living in the same household (35 dog-dog dyads) and 29 with their owner (29 dog-owner dyads) (for more see Supplementary Methods). All dyads were tested outdoors, in an experiment including four episodes: exploration of an unfamiliar environment, separation from the partner, reunion with the partner and a novel object test.

In order to reduce the number of variables coded from both the dog-human and the dog-dog interactions, we conducted a Principal Component Analysis (PCA) that extracted three behavioral components: Reference to partner (explaining 22.86% of the variance and including Alternation of gaze between the partner and the novel object, Gaze at the partner, Greeting, Fear-related behaviors), Affiliation (explaining 16.47% of the variance and including Affiliative behaviors, Synchronized behaviors and Play), and Stress (explaining 13.70% of the variance and including Stress-related behaviors and Marking), Supplementary Table S2.

To characterize the intra- and inter-specific relationship type that each individual had with each of its partners, we ran two Cluster Analyses (one using data from the dog-dog tests and one using data from the dog-owner tests, in order to be able to compare intra- vs. inter-specific relationship types in later analyses, see below) on the three components extracted from the PCA (Reference, Affiliation, Stress) and, for further validation, a Discriminant Function Analysis (see Supplementary Results R6).

Three clusters emerged in relation to the dog-dog dyads. The first cluster included dogs that showed high Reference, medium Affiliation and high Stress (“Insecure dog-dog relationship”, N = 34, Supplementary Tables S3 and S5). The second cluster contained dogs showing little Reference, high Affiliation, and mild Stress (“Friend dog-dog relationship”, N = 22, Supplementary Tables S3 and S5). The third cluster included dogs that showed low Reference, low Affiliation, and low Stress (“Independent dog-dog relationship”, N = 14, Supplementary Tables S3 and S5). Amongst the 24 dogs tested with two conspecific partners, 13 showed the same behavioral pattern towards both conspecific partners, while 11 showed different behavioral patterns to their two partners.

Two clusters emerged in relation to the dog-owner dyads. Relative to each other, the first cluster (N = 19, Supplementary Tables S4 and S5) included dogs showing lower Reference, lower Affiliation and higher Stress (“Tense dog-owner relationship”) than the second dog-owner cluster (“Close dog-owner relationship”) (N = 10, Supplementary Tables S4 and S5).

**Figure 1:**
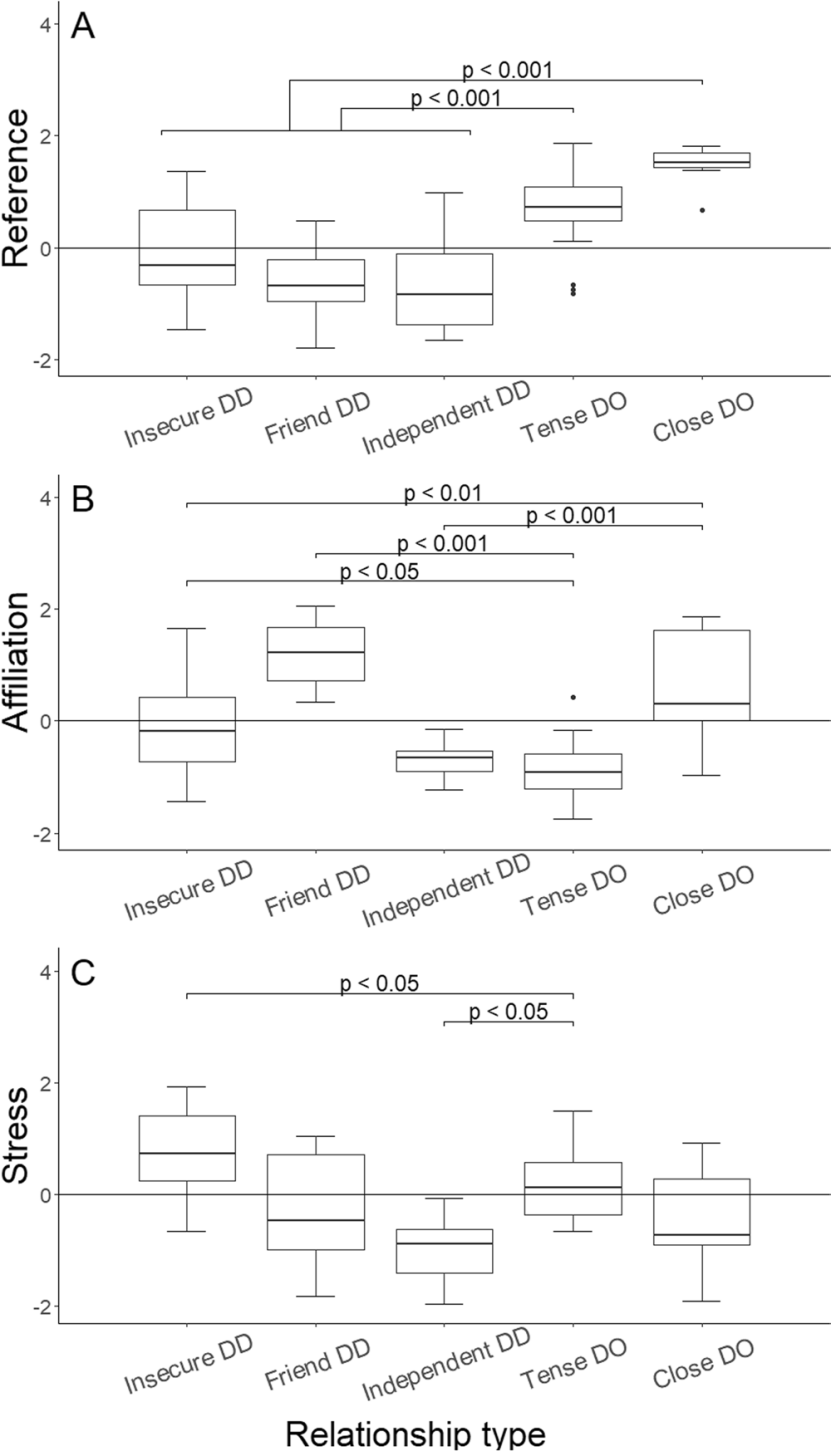
Levels of Reference, Affiliation and Stress shown in each relationship type. Median and interquartile range (IQR; represented by the box), 25^th^ percentile + 1.5 IQR, and 75^th^ − 1.5 IQR (represented by the lower and the upper whiskers respectively) of each behavioral component extracted by the PCA according to clusters classification. On the y-axis, scores obtained by each behavioral variable extracted by the PCA (A = Reference; B = Affiliation; C = Stress). On the x-axis, dog-dog and dog-owner clusters classification. Abbreviations: DO = dog-owner relationship type; DD = dog-dog relationship type.

To compare the two dog-owner relationship clusters to the three dog-dog relationship clusters, we ran linear mixed models using the behavioral components extracted in the PCA as dependent variables and the cluster classifications for the dog-dog and dog-owner dyads, breed, behaviors directed to the owner in the dog-dog tests (i.e. gaze and being in close proximity), and order of testing as predictors, as well as subject, partner, dyad, and household as random factors. We found that dogs in both dog-owner clusters showed higher Reference than dogs in any of the three dog-dog clusters (“Close” dog-owner cluster different from: “Friend”: estimate ± s.e.m. = 2.07 ± 0.38, t = 5.46, p < 0.001, “Insecure”: estimate ± s.e.m. = 1.64 ± 0.36, t = 4.50, p < 0.001, and “Independent”: estimate ± s.e.m. = 2.24 ± 0.40, t = 5.59, p < 0.001; “Tense” dog-owner cluster different from: “Friend”: estimate ± s.e.m. = 1.51 ± 0.26, t = 5.75, p < 0.001, “Insecure”: estimate ± s.e.m. = 1.08 ± 0.25, t = 4.40, p < 0.001, and “Independent”: estimate ± s.e.m. = 1.68 ± 0.30, t = 5.64, p < 0.001; Fig. 1A). Beyond this, in regard to Affiliation, dogs belonging to the “Close” dog-owner cluster showed no difference to the “Friends” dog-dog cluster (estimate ± s.e.m. = −0.30 ± 0.36, t = −0.83, p > 0.05) whereas they showed higher Affiliation than the “Insecure” (estimate ± s.e.m. = 1.18 ± 0.34, t = 3.45, p = 0.005, Fig. 1B) and “Independent” dog-dog clusters (estimate ± s.e.m. = 1.71 ± 0.37, t = 4.59, p < 0.001, Fig. 1B). On the other hand, dogs belonging to the “Tense” dog-owner cluster showed lower Affiliation than the “Friend” (estimate ± s.e.m. = −2.09 ± 0.24, t = −8.74, p < 0.001, Fig. 1B) and the “Insecure” dog-dog clusters (estimate ± s.e.m. = −0.61 ± 0.22, t = −2.77, p = 0.04, Fig. 1B), and similar levels of Affiliation to the “Independent” dog-dog cluster (estimate ± s.e.m. = −0.09 ± 0.27, t = −0.33, p > 0.05, Fig. 1B). Moreover, dogs of the “Close” dog-owner cluster showed comparable levels of Stress to all dog-dog clusters (“Friend”: estimate ± s.e.m. = 0.49 ± 0.37, t = 1.31, p > 0.05, “Insecure”: estimate ± s.e.m. = −0.57 ± 0.36, t = −1.59, p > 0.05, and “Independent”: estimate ± s.e.m. = 0.97 ± 0.39, t = 2.47, p = 0.09, Fig. 1C), while dogs belonging to the “Tense” dog-owner cluster showed more Stress than the dogs belonging to the “Independent” dog-dog cluster (estimate ± s.e.m. = 0.82 ± 0.30, t = 2.78, p = 0.04, Fig. 1C), less Stress than the dogs belonging to the “Insecure” dog-dog cluster (estimate ± s.e.m. = −0.72 ± 0.24, t = −2.95, p = 0.03, Fig. 1C), and similar levels of Stress than the dogs belonging to the “Friend” dog-dog cluster (estimate ± s.e.m. = 0.34 ± 0.26, t = 1.29, p > 0.05, Fig. 1C). Neither the breed of the subject, how many times it had previously been tested nor its contact-seeking to the owner in the dog-dog tests had a major influence on the behavioral variables here analyzed (Supplementary Results R4 and R5).

### Reaction to a social threat in presence of partners with different relationships

**Figure 2:**
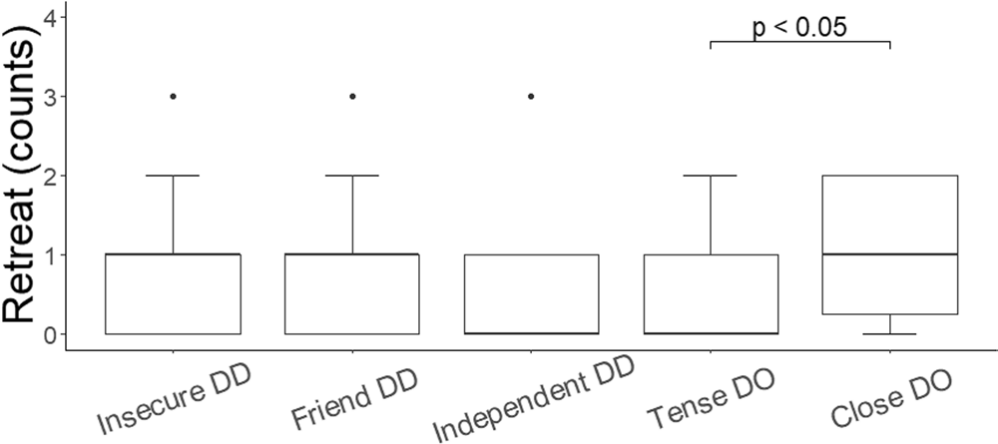
Counts of Retreat shown during the Social Threat test by individuals belonging to each relationship type. Median and interquartile range (IQR; represented by the box), 25^th^ percentile + 1.5 IQR, and 75^th^ − 1.5 IQR (represented by the lower and the upper whiskers respectively) of the variable Retreat according to clusters classification. On the x-axis, dog-dog and dog-owner clusters classification. Abbreviations: DO = dog-owner relationship type; DD = dog-dog relationship type.

**Figure 3:**
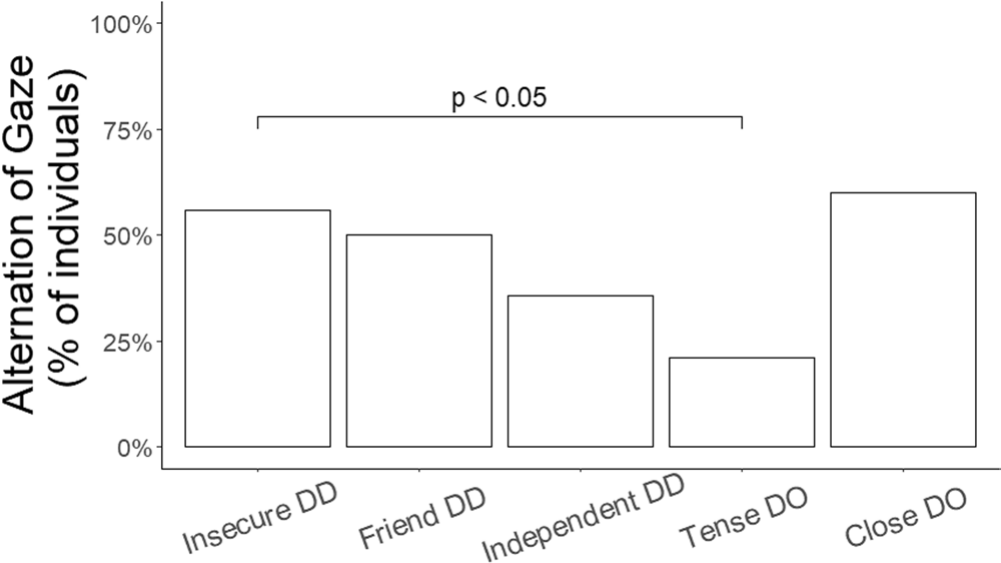
% of individuals alternating the gaze between the partner and the experimenter during the Social Threat test according to cluster classification. On the x-axis, dog-dog and dog-owner clusters classification. Abbreviations: DO = dog-owner relationship type; DD = dog-dog relationship type.

The dyads tested in the Relationship test were then tested in a final test that combined elements from the “Threatening approach” test^23,24^ and the “Ghost” test used in the Dog Mentality Assessment^25^. This so-called “Social Threat” test presented a social threat, where an unfamiliar person approached the dyad in a threatening manner whilst wearing a mask and a costume. This test was used to assess the functional value of the partner in shaping the reaction of the subject facing a threatening situation. We coded six behavioral variables during the “Social Threat” (i.e. Retreat, Alternation of gaze between the partner and the experimenter, Friendly, Aggression, Passive, and Hide behind the partner; see Supplementary Table S1 for definitions). We found that dogs belonging to the “Close” dog-owner cluster retreated more often than dogs in the “Tense” dog-owner cluster (estimate ± SE = 0.71 ± 0.23, z = 3.15, p = 0.01, Fig. 2) and that dogs of the “Insecure” dog-dog cluster tended to retreat more often than dogs belonging to the “Tense” dog-owner cluster (estimate ± SE = 0.46 ± 0.17, z = 2.65, p = 0.06, Fig. 2). Similarly, we found that dogs belonging to the “Tense” dog-owner cluster were less likely to alternate their gaze between the experimenter and the partner than dogs belonging to the “Insecure” dog-dog cluster (estimate ± SE = −8.20 ± 2.76, z = −2.97, p = 0.02, Fig. 3). Cluster classification did not have a significant effect on the likelihood of showing a friendly (X2(4) = 19.20, p > 0.05) or an aggressive reaction (X2(4) = 2.61, p > 0.05), or on the likelihood of remaining passive (X2(4) = 3.45, p > 0.05). Moreover, since only 15 dogs hid behind the partner (N = 5 “Tense” dog-owner cluster, N = 4 “Insecure” dog-dog cluster, N = 4 “Friend” dog-dog cluster, N = 2 “Independent” dog-dog cluster) and none of those belonged to the “Close” dog-owner relationship type, we could not run a statistical analysis using this variable as response.

## Discussion

In the present study, the bottom-up approach we adopted revealed that dogs’ interactions with their conspecific and human partners could be described in terms of reference/information seeking (described by former studies mostly in dog-human interactions)^26^, affiliation (mostly investigated in dog-dog interactions)^18,22,27^ and stress alleviation (mostly investigated in dog-human interactions^13,28,29^ but see also^30^), bringing together relationship components that to date have been investigated only singularly (either at an intra- or at an inter-specific level) and not in a comparative way.

Based on these components, the present study allowed the categorization of five different relationship types that dogs form with their owners and household dogs. These relationship types can be described as: a close dog-owner relationship characterized by the highest levels of reference and affiliation (comparable to the affiliation level shown in the friend-type dog-dog relationship), and the lowest levels of stress (as low as in the independent dog-dog relationship). This cluster seems to describe dogs that have an affiliative relationship with their owner, whom they attended to and could use for stress alleviation. On the other hand, we also identified a tense dog-owner relationship characterized by higher levels of reference than the other dog-dog clusters (but still lower than a close dog-owner relationship), but lower levels of affiliation than some of the dog-dog relationship types (i.e. friend and insecure), and higher levels of stress than the independent dog-dog cluster. This cluster seems to describe dogs with an insecure, tense relationship with their owners, who are hardly seen as affiliative partners and appear to function as poor stress buffers.

As intra-specific relationship types, we identified a friend dog-dog relationship (based on medium reference, high affiliation, and low stress), an insecure dog-dog relationship (characterized by medium reference, medium affiliation, and high stress), and an independent dog-dog relationship (showing low reference, low affiliation, and low stress). Interestingly, almost half of the subjects tested with at least two dog partners had different relationships with their different partners, suggesting that the behavioral patterns identified here were modulated by the partner’s identity and cannot be entirely attributed to the individual’s personality.

The results obtained from the comparison of these relationship types show that dog-owner relationships in general differ from dog-dog relationships only in terms of reference but not regarding affiliation and stress. Notably, mainly gazing behaviors (total gaze duration and alternation of gaze between the partner and the novel object) loaded into the reference component. These results are in line with previous studies showing that dogs pay more attention to a human demonstrator than to a conspecific one^31^. One may argue that this increased gazing is a behavioral expression of the unique dog-owner relationship, as suggested by^3^. However, it is also possible that pet dogs are more attentive to their owners as a result of their previous history of reinforcement (i.e. learnt to face their owners throughout their training and/or their everyday social interactions with the owner)^32–35^. This because owners (and humans in general) might be more responsive to gaze (and to visual communication in general) than dogs. Pet dogs might also look more at their owners than at other dogs because they may see their owners as partners that are more likely to tell them what to do (compared to a dog partner) in the specific context they were tested in (i.e. fenced area in an urbanized environment), and/or as partners from whom to acquire information regarding the novel environment and novel stimuli they were facing (e.g. during the Novel Object test)^26^. Additionally, it cannot be excluded that in the present setting, dog owners might have behaved differently from usual because of being observed and video-recorded, triggering an increase in their dogs’ attentiveness. Finally, one may suggest that in the current study the subjects’ gazing at their dog partners was reduced by the owners who were, even if quietly and unobtrusively, standing outside the enclosure and, thus, may have attracted the subjects’ attention. Having the owners present during the dog-dog tests was unavoidable, as previous studies had shown that when two dogs from the same household are tested in an unfamiliar situation, their owner’s absence may prevent them from showing their natural social interactions towards one another^36,37^. Here we explicitly checked whether time spent looking at the owner and in his/her proximity influenced the dogs’ behavior and found no effect of this variable.

Regarding the other two components, affiliation and stress, we found that dogs do not affiliate more with humans than with conspecifics and they do not benefit from their owners’ presence more than conspecifics in terms of stress alleviation. Considering the potential limitations of the reference component explained above, we suggest that in the current study affiliation and stress are more informative when comparing the intra- and interspecific relationships of pet dogs. Based on these two components, our results suggest that dogs form similar relationships with both humans and dogs, and that, with both kinds of partners, the quality of the bond varies more with the individual partner than between dog vs. human partners. As such, both with the owners and with other dogs some relationship types were characterized by either high affiliation and low stress (friend dog-dog and close dog-owner), low affiliation and high stress (insecure dog-dog and tense dog-owner) or low affiliation and low stress (independent dog-dog). Therefore, dogs affiliated more with the owner than with a dog companion only if they had a close relationship with him/her but, even in this case, only if they had an insecure or independent relationship with the dog partner. Towards their “dog friends”, dogs showed a similar amount of affiliation as towards the owners with whom they had a close relationship. In contrast, when dogs had a tense relationship with their owner, they affiliated less with her/him than with a companion dog, not only when it was a friend but even when they had a more insecure relationship with it.

Stress alleviation has mostly been investigated in dog-human interactions, although at least in one case, a positive association between social support and affiliative relationships have been already identified in an intraspecific setting^27^. In the current study, however, it is not possible to disentangle whether the stress-related behaviors shown by the subjects reflected the overall stress response to the environmental context (alleviated more or less by the presence of the partner) or whether eventually the presence of the partner itself may evoke further stress. The findings that dogs in a tense relationship with their owner showed more stress than dogs behaving rather independently from their dog partner, and that dogs in an insecure relationship with their dog companion showed even more stress that dogs tested with a tense owner, seem to indicate that these two kinds of partners may have increased the subjects’ stress level. Importantly, we do not suggest that dogs in these cases would be afraid of their partners. In fact, in this study, fear-related behaviors loaded on another component, i.e. reference, most likely because these behaviors were evoked by specific parts of the experimental procedure, e.g. the novel object appearing. This can also explain why the amount of fear-related behaviors was correlated with gazing at the partner but not with the amount of stress-related behaviors that, presumably, was affected rather by the subjects’ overall perception of the experimental context. With the subjects having had negative experiences with their partners showing inadequate or even competitive behaviors in earlier challenging or stressful situations, the presence of such partners might have contributed to perceiving the entire context more stressful. In line with this, former studies have shown that even in a potentially positively-valenced situation like play, a tense relationship with the partner might increase a dog’s stress levels^38^.

Future studies will need to investigate whether and when partners with different relationships can help or hinder dogs cope with stress. Nevertheless, what emerged here is that dogs were not overall more stressed when tested with their conspecific than with their human partners (contrary to^30^). Rather the relationship with the partner present (no matter the species the partner belonged to) modulated their reaction. In particular, both human and dog partners may have stress alleviating or stress increasing effect, depending on the individual relationship the subject has with them.

Further support for the role of the relationship with the partner in dealing with challenging situations comes from our social threat test. Here we aimed to test whether the type of relationship with the partner present could influence dogs’ reaction to a threatening stimulus. We found no major difference between dogs tested with the owner or with a conspecific partner, suggesting that, also in this case, partner species might not play a strong role in shaping the reactions of dogs to such situations. Confirming this, we also found that dogs alternate their gaze not only between a human partner and a scary stimulus, but display this behavior with conspecific partners as well, and might do so even more often than with an owner with whom they have a tense relationship. This behavior has been used as the main indicator of social referencing^39^, and was to date mostly described when dogs were accompanied by a human^26^. Interestingly, however, it appears that such a social threat test may be more suited to test the functionality of pet dogs’ human-directed than their intraspecific relationships, as we found no difference in the reactions of dogs across the three dog-dog relationship types, whereas dogs belonging to the close dog-owner relationship type were more likely to retreat than dogs having a tense relationship with the owner. These later results are in line with a previous study, in which dogs’ reaction to a socially stressful situation was associated with low levels of warmth and enthusiasm showed by the owners^19^ and confirm the role of the dog-owner relationship in shaping the dogs’ reaction to a stressful event (as already shown in humans and non-human animals)^40,41^. Furthermore, the fact that dogs were less likely to retreat from the threatening experimenter when accompanied by the least affiliative partners (i.e. tense dog-owner and independent dog-dog relationships), is in line with what has already been shown in a previous study demonstrating that dogs are more likely to join inter-group conflicts when affiliative partners are present^27^.

Overall, the main finding of the present study is that individual partners play a more important role in shaping dogs’ relationships than the species the partner belongs to. This suggests that, at least in terms of affiliation and stress-coping, the relationships dogs build with humans are not necessarily special and unique (as claimed in^3,5^), rather the ability to form enduring, affiliation-based bonds that can socially modulate coping with stressful situations is also present in dogs’ intra-specific relationships. In support to this, it has been shown that also adults of other species can form affiliative bonds with peers that help them cope with stress (see^42^ for a review). Future studies could, for example, investigate whether also wolves, the closest relatives of dogs, show similar strong affiliative and stress-buffering relationships with conspecifics. If this were the case, then dogs might have inherited the ability to form these kinds of relationships from their common ancestor, and not acquired it during the process of domestication^6^.

These results have major practical relevance since pet dogs do not have the possibility to choose their social partners (either human or conspecific). Being obliged to engage in non-optimal relationships can increase dogs’ chronic stress levels and, consequently, reduce their welfare. Social chronic stress has, in fact, been associated with a suppressed immune system^43^ and with various disruptive behaviors (e.g. aggression, see^44^ for a review), highlighting the importance of the social environment. With the present study we made the first step in understanding how relationships influence dogs’ response to stress, and our results could provide the basis for future studies aimed at investigating how relationships ultimately affect dogs’ health, behavioral problems, and human safety.

It is important to note that the present study was conducted on a relatively limited sample of dogs originating from a rather homogeneous population, that is, pet dogs living in an urban environment. Although the choice of this population was necessary to allow for a direct comparison between intra- and inter-specific relationships, future studies would need to investigate to what extent these results are generalizable to other populations of dogs (e.g. living as free-ranging dogs without human supervision). Moreover, the relationship components identified here reflect the specific sub-tests used in the present study. Future studies might expand on the quantity and quality of contexts (e.g. controlled encounter with an unfamiliar dog) in which the intra- and interspecific interactions are analyzed, which may lead to the emergence of additional relationship components.

To conclude, our results present evidence that conspecifics might be as good partners as humans in respect to affiliation and social modulation of stress, but there might still be a difference in regard to reference and information seeking. Future research would need to investigate to what extent the different relationship components identified here contributed to the evolutionary success of the domestic dog.

## Methods

### Subjects

Dog owners (12 F/2 M, mean age ± s.d. = 34.6 ± 10.01 years) were recruited from the database of the Clever Dog Lab (Vienna, Austria). A total of 48 dogs were involved in the present study (31 F (16 neutered)/17 M (6 neutered), mean age ± s.d. = 77.62 ± 44.94 months). They were mainly pure-bred Border Collies and mongrels (23 Border Collies, 15 mongrels, 4 Shetland Sheepdogs, 2 Miniature Pinschers, 1 Mudi, 1 Berger des Pyrenees, 1 Kelpie, 1 German Shepherd). They lived in 14 different multiple dog households composed of at least 3 dogs (range between 3 and 7 dogs per household). The subjects lived in that household and with the conspecific partners involved in the study for at least one year before the onset of the experiment. Members of none of the dog dyads were related, except one pair (Kandy mother of Connor).

### Procedures

Testing took place in outdoor areas (Area A: 24 × 13 m; Area B: 17 × 19 m, Area C: 25 × 13 m) on the campus of the University of Veterinary Medicine, Vienna (Austria) between July 2014 and July 2015. The areas were delimited by not opaque chain-link fences. Trees were present in the areas and the ground was covered with grass. The surroundings were quiet, and passers-by were far away enough for the subjects to be undisturbed. See Fig. S1 for a schematic representation of one of testing areas.

Each experiment involved a dyad composed of a dog and its owner or 2 dogs living in the same household. Each subject was tested with the owner and/or with one or two different conspecific partners (see Supplementary Methods for more). Each dyad participated in the “Relationship test” (consisting of 4 sub-tests conducted in the same order for all subjects: exploration of an unfamiliar outdoor area, short physical separation from the partner, subsequent reunion, novel object test) and in the “Social Threat” test.

Relationship test:

For dog-owner dyads:

- Exploration (3 minutes): the owner and the dog entered the outdoor area and the owner released the dog from the leash while the experimenter closed the gate behind them. The owner was instructed to behave as similar as possible to visiting a dog park and he/she was asked to randomly walk around the fenced area without ever calling the dog. The owner was allowed to respond to any attention soliciting by the dog (by looking or talking to the dog) but was not allowed to give commands to the dog or to interrupt the behavior the dog showed during the test.
- Separation (3 minutes): at the end of the Exploration phase, the dog was accompanied by the owner to a smaller area covered with an opaque fence which allowed visual separation from the owner. Once the owner was out of view from the subject and seated on a chair on the opposite side of the enclosure, the Separation phase started.
- Reunion (3 minutes): following the separation, the experimenter opened the door to the small enclosed area and the dog could meet the owner again. The owner stood up and moved few steps from where the chair was placed in order to be visible again for the dog. The owner was instructed not to call the dog but to respond to the greeting of the dog as he/she would normally do. Furthermore, the owner was asked not to re-initiate the greeting if the dog stopped interacting with him/her, rather to start to behave again as in the Exploration phase. After the 3 minutes passed, the owner put the dog on leash, and they went to a location from where the dog could not witness what was happening in the enclosure.
- Novel Object (3 minutes): while the dog and the owner were outside the enclosure for about 5 minutes, the experimenter placed a novel object (e.g. plastic cube) dangling on a rope hanging from a branch of a tree located in the enclosure. The free extremity of the rope was held by the experimenter who was standing out of sight outside the enclosure and manipulated the rope so that the novel object moved up and down during the test. Once the experimenter was in position, the owner was allowed to enter the enclosure again with the dog. The Novel Object phase started when the dog was released from the leash into the area. The owner was asked to behave as in the Exploration phase. After the 3 minutes passed, the owner put the dog on the leash and left the enclosure.
  For dog-dog dyads:
  The procedure was the same as for the dog-owner test with the only difference that:
- The two dogs were released in the enclosure by the owner who then stood outside the enclosure, close to the entrance, ignoring the dogs (Exploration, Novel Object).
- During the separation phase the two dogs were placed in the two smaller areas without the possibility of seeing each other but both being able to see the owner standing outside the enclosure.

Social Threat test (30 seconds):

After about 5 minutes from the end of the novel object test, the owner released the two dogs into a smaller fenced area (2 m × 4 m) inside the experimental enclosure or entered it together with her/his dog. From a distance of 10 m an experimenter showed up wearing a costume which also covered her face and approached them walking with exaggerated movements (e.g. jumping up or agitating the arms). She approached the small fenced area to a distance of 1 m alongside which she walked for 5 secs till she turned her back and then left the enclosure.

Whenever a dog was tested more than once, the experiment was conducted in a different fenced area, a different object was used in the Novel Object test, and the experimenter wore a different costume for the “Social Threat” test (for a total of 4 costumes, all having a humanoid shape: i.e. a dragon, an alien, a vampire, a Death reaper). The order of partners, the different areas, novel objects, and costumes were counterbalanced across subjects.

#### Behavioral analysis

The experiments were video recorded by two experimenters standing outside the enclosure with two hand-held cameras. The videos of the two cameras were then merged using an in-house program and then analyzed off-line using Solomon coder (copyright © 2006–2017 by András Péter). See Supplementary Table S1 for a complete list of the behaviors analyzed throughout the experiment. All videos were coded by one main coder, while a second coder coded 20% of the randomly chosen videos in order to calculate inter-observer reliability. Inter-observer reliability was good or excellent depending on the coded variable (Intraclass Correlation Coefficient, ICC, ranging between 0.64 and 0.97).

### Statistical analysis

In order to reduce the number of variables included in the present study, we ran a Principal Component Analyses (PCA) with Varimax rotation on the behavioral variables coded during the Relationship test. We checked sphericity using Bartlett’s test and we calculated the KMO (R package *psych*). Furthermore, we calculated internal consistency of each extracted component by calculating Cronbach’s alpha. In order to analyze whether dogs showed different behavioral patterns with the owner or with conspecifics, we ran a Discriminant Function Analysis (DFA, R package *MASS*; Supplementary Results R1).

Additionally, to classify the individuals based on their behavior shown with different partners and, thereby, to define categories of social relationships, we conducted cluster analyses separately for dog-dog and dog-human on the components extracted from the PCA. Since the second aim of the study was to compare intra- and inter-specific relationships dogs build in their social group, we conducted separated cluster analyses on dogs when tested either with a conspecific or with their owner. Visual examination of the dendrogram revealed the number of clusters to be extracted, which were then assigned using k-means cluster analysis with Euclidean distance as dissimilarity measure. The solutions were additionally validated using Discriminant Function Analysis (DFA, R package *MASS*; Supplementary Results R1). After that, in order to test whether the relationships dogs build with conspecifics and with the owner are different, we ran linear mixed models (LMMs) using the behavioral components extracted in the two PCAs as response variables, cluster classification of the relationships, breed of the dog (2-level categorical variable: Border Collie vs. other breeds), session (ordinal variable ranging from 1 to 3), and the variable “Owner” (see Supplementary Results R4) as predictors, as well as subject, partner, dyad, and household as random factors (R package *nlme*). We then compared specifically chosen clusters (the three dog-dog clusters vs. the two dog-owner clusters) using post-hoc TukeyHSD weighted contrasts (R package *multcomp*).

Moreover, in order to test whether the relationship with the partner present affected how a dog would react during the “Social Threat” test, we ran Generalized Linear Mixed Models (GLMMs) using the behavioral components coded during this test as response variables (binomial distribution for all variables, beside “Retreat” that had a poisson distribution), clusters classification of the relationships, breed of the dog (2-level categorical variable: Border Collie vs. other breeds), and session (ordinal variable ranging from 1 to 3) as predictors, as well as subject, partner, dyad, and household as random factors (R package *nlme*). We then compared all pairs of clusters using post-hoc TukeyHSD weighted contrasts (R package *multcomp*).

Homogeneity of variance and the normality of residuals was checked using quantile-quantile plots and by plotting residuals against fitted values. Inter-observer reliability was assessed calculating Intraclass Correlation Coefficients for each behavioral variable (ICC, R package *irr*). All statistical analyses were conducted using the software SPSS v. 24 (IBM, Chicago IL) and R (version: 3.4.4, R Core Team 2018).

### Ethic statement

The experimental procedures were approved in accordance with GPS guidelines and national legislation by the Ethical Committee for the use of animals in experiments at the University of Veterinary Medicine Vienna (Ref: 19/04/97/2014). All owners provided a written informed consent prior to the onset of the experiment.

## Supplementary information

Supplementary Information

## Data Availability

The datasets generated during and/or analyzed during the current study are available from the corresponding author on request.

## References

[1] Ferreira, J. P., Leitao, I., Santos-Reis, M. & Revilla, E. Human-related factors regulate the spatial ecology of domestic cats in sensitive areas for conservation. *PLoS One*, 10.1371/journal.pone.0025970 (2011). 10.1371/journal.pone.0025970 PMC3197152 22043298

[2] Hughes J, Macdonald DW (2013). A review of the interactions between free-roaming domestic dogs and wildlife. Biol. Conserv..

[3] Miklósi Á, Topál J (2013). What does it take to become ‘best friends’? Evolutionary changes in canine social competence. Trends Cogn. Sci..

[4] Hare B, Tomasello M (2005). Human-like social skills in dogs?. Trends Cogn. Sci..

[5] Nagasawa M (2015). Oxytocin-gaze positive loop and the coevolution of human-dog bonds. Science (80-.)..

[6] Range F, Virányi Z (2015). Tracking the evolutionary origins of dog-human cooperation: the Canine Cooperation Hypothesis. Front. Psychol..

[7] McGreevy PD, Masters AM (2008). Risk factors for separation-related distress and feed-related aggression in dogs: Additional findings from a survey of Australian dog owners. Appl. Anim. Behav. Sci..

[8] Berman M, Dunbar I (1983). The social behaviour of free-ranging suburban dogs. Appl. Anim. Ethol..

[9] Boyko AR (2009). Complex population structure in African village dogs and its implications for inferring dog domestication history. Proc. Natl. Acad. Sci. USA.

[10] Cafazzo S, Valsecchi P, Bonanni R, Natoli E (2010). Dominance in relation to age, sex, and competitive contexts in a group of free-ranging domestic dogs. Behav. Ecol..

[11] Boitani L, Ciucci P (1995). Comparative social ecology of feral dogs and wolves. Ethol. Ecol. Evol..

[12] Hare B, Brown M, Williamson C, Tomasello M (2002). The domestication of social cognition in dogs. Science.

[13] Topál J, Miklósi Á, Csányi V, Dóka A (1998). Attachment behavior in dogs (Canis familiaris): A new application of Ainsworth’s (1969) Strange Situation Test. J. Comp. Psychol..

[14] Prato-Previde E, Custance DM, Spiezio C, Sabatini F (2003). Is the dog-human relationship an attachment bond? An observational study using Ainsworth’s strange situation. Behaviour.

[15] Topál J (2005). Attachment to humans: a comparative study on hand-reared wolves and differently socialized dog puppies. Anim. Behav..

[16] Cafazzo, S., Bonanni, R., Valsecchi, P. & Natoli, E. Social variables affecting mate preferences, copulation and reproductive outcome in a pack of free-ranging dogs. *PLoS One* **9** (2014). 10.1371/journal.pone.0098594 PMC4048177 24905360

[17] Dale R, Range F, Stott L, Kotrschal K, Marshall-Pescini S (2017). The influence of social relationship on food tolerance in wolves and dogs. Behav. Ecol. Sociobiol..

[18] Marshall-Pescini S, Schwarz JFL, Kostelnik I, Virányi Z, Range F (2017). Importance of a species’ socioecology: Wolves outperform dogs in a conspecific cooperation task. Proc. Natl. Acad. Sci..

[19] Cimarelli G, Turcsán B, Bánlaki Z, Range F, Virányi Z (2016). Dog Owners’ Interaction Styles: Their Components and Associations with Reactions of Pet Dogs to a Social Threat. Front. Psychol..

[20] Horn L, Range F, Huber L (2013). Dogs’ attention towards humans depends on their relationship, not only on social familiarity. Anim. Cogn..

[21] Bonanni R, Cafazzo S, Valsecchi P, Natoli E (2010). Effect of affiliative and agonistic relationships on leadership behaviour in free-ranging dogs. Anim. Behav..

[22] Trisko RK, Sandel AA, Smuts B (2016). Affiliation, dominance and friendship among companion dogs. Behaviour.

[23] Vas J, Topál J, Gácsi M, Miklósi Á, Csányi V (2005). A friend or an enemy? Dogs’ reaction to an unfamiliar person showing behavioural cues of threat and friendliness at different times. Appl. Anim. Behav. Sci..

[24] Vas J, Topál J, Győri B, Miklósi Á (2008). Consistency of dogs’ reactions to threatening cues of an unfamiliar person. Appl. Anim. Behav. Sci..

[25] Svartberg K, Forkman B (2002). Personality traits in the domestic dog (Canis familiaris). Appl. Anim. Behav. Sci..

[26] Merola I, Prato-Previde E, Marshall-Pescini S (2012). Social referencing in dog-owner dyads?. Anim. Cogn..

[27] Bonanni R, Valsecchi P, Natoli E (2010). Pattern of individual participation and cheating in conflicts between groups of free-ranging dogs. Anim. Behav..

[28] Csoltova E, Martineau M, Boissy A, Gilbert C (2017). Behavioral and physiological reactions in dogs to a veterinary examination: Owner-dog interactions improve canine well-being. Physiol. Behav..

[29] Schöberl I (2016). Social factors influencing cortisol modulation in dogs during a strange situation procedure. J. Vet. Behav. Clin. Appl. Res..

[30] Tuber DS, Sanders S, Hennessy MB, Miller JA (1996). Behavioral and glucocorticoid responses of adult domestic dogs (Canis familiaris) to companionship and social separation. J. Comp. Psychol..

[31] Range F, Horn L, Bugnyar T, Gajdon GK, Huber L (2009). Social attention in keas, dogs, and human children. Anim. Cogn..

[32] Bentosela M, Barrera G, Jakovcevic A, Elgier AM, Mustaca AE (2008). Effect of reinforcement, reinforcer omission and extinction on a communicative response in domestic dogs (Canis familiaris). Behav. Processes.

[33] Jakovcevic A, Elgier AM, Mustaca AE, Bentosela M (2010). Breed differences in dogs’ (Canis familiaris) gaze to the human face. Behav. Processes.

[34] Passalacqua C (2011). Human-directed gazing behaviour in puppies and adult dogs, Canis lupus familiaris. Anim. Behav..

[35] Wallis LJ (2015). Training for eye contact modulates gaze following in dogs. Anim. Behav..

[36] Mariti C (2017). Intraspecific relationships in adult domestic dogs (Canis familiaris) living in the same household: A comparison of the relationship with the mother and an unrelated older female dog. Appl. Anim. Behav. Sci..

[37] Mariti C, Carlone B, Ricci E, Sighieri C, Gazzano A (2014). Intraspecific attachment in adult domestic dogs (Canis familiaris): Preliminary results. Appl. Anim. Behav. Sci..

[38] Horváth Z, Dóka A, Miklósi Á (2008). Affiliative and disciplinary behavior of human handlers during play with their dog affects cortisol concentrations in opposite directions. Horm. Behav..

[39] Russell CL, Bard KA, Adamson LB (1997). Social referencing by young chimpanzees (Pan troglodytes). J. Comp. Psychol..

[40] Koolhaas J (1999). Coping styles in animals: current status in behavior and stress-physiology. Neurosci. Biobehav. Rev..

[41] Hostinar CE, Sullivan RM, Gunnar MR (2014). Psychobiological mechanisms underlying the social buffering of the hypothalamic–pituitary–adrenocortical axis: A review of animal models and human studies across development. Psychol. Bull..

[42] Hennessy MB, Kaiser S, Sachser N (2009). Social buffering of the stress response: Diversity, mechanisms, and functions. Front. Neuroendocrinol..

[43] Dhabhar FS (2014). Effects of stress on immune function: The good, the bad, and the beautiful. Immunol. Res..

[44] Tielbeek, J. J. *et al*. The impact of chronic stress during adolescence on the development of aggressive behavior: A systematic review on the role of the dopaminergic system in rodents. *Neurosci. Biobehav. Rev*, 10.1016/j.neubiorev.2016.10.009 (2016). 10.1016/j.neubiorev.2016.10.009 27826069

