# Supplementary material for: Pet dogs’ relationships vary rather individually than according to partner’s species
Source: Sci Rep. 2019 Mar 5;9:3437. doi: 10.1038/s41598-019-40164-x (PMC6401312; doi:10.1038/s41598-019-40164-x)
Supplement: Supplementary file 1 — Supplementary Information [file 41598_2019_40164_MOESM1_ESM.docx]

Manuscript Title: Pet dogs' relationships vary rather individually than according to partner’s species

**Authors**

G. Cimarelli^1,2,^*, S. Marshall-Pescini^2,1^, F. Range^2,1^, Z. Virányi^1^

^1^Comparative Cognition, Messerli Research Institute, University of Veterinary Medicine Vienna, Medical University of Vienna, University of Vienna, Austria.

^2^ Domestication Lab, Konrad Lorenz Institute of Ethology, University of Veterinary Medicine Vienna, Austria.

*Corresponding author: Giulia Cimarelli (GC), ORCID: 0000-0002-6031-0364, e-mail: giulia.cimarelli@vetmeduni.ac.at

Supplementary Information (SI)

Supplementary Tables:

Table S1: Behavioral variables coded in the Relationship and the Social Threat tests.

Table S2: Rotated component matrix restricted to three components based on behavior showed in the Relationship test.

Table S3: Clusters of dogs tested with a conspecific partner.

Table S4: Clusters of dogs tested with the owner.

Table S5: Clusters and raw behavioral variables.

Table S6: Linear Mixed Models (LMMs) and Generalized Linear Mixed Models (GLMMs) schematic results.

| **Table S1. Behavioral variables coded in the present study** | | | | | |
| --- | --- | --- | --- | --- | --- |
| **Behavior** | **Type** | **Definition** | **Subtest** | **Transformation (% of dogs falling in each category)** | **Inter-rater reliability** |
| Gaze at the partner | Duration | The dog orients its head towards the partner | Pulled for Exploration + Reunion + Novel Object | [0.0, 27.0] sec = 0 (33.3%)  [27.1, 61.0] sec = 1 (33.3%)  [61.1, 360] sec = 2 (33.3%) | F(37) = 21.3, p < 0.001, ICC = 0.88 |
| Alternation of gaze between the partner and the novel object | Count | The dog´s head orientation towards the partner is followed/preceded within 1 s by a look towards the object | Novel Object | 0 = 0 (41.4%)  [1,3] = 1 (31.3%)  [4,51] = 2 (27.3%) | F(37) = 9.8, p < 0.001, ICC = 0.82 |
| Affiliative behaviors | Count | The dog grooms, sniffs, body rubs or licks the partner | Pulled for Exploration + Reunion + Novel Object | [0] = 0 (68.7%)  [1,4] = 1 (31.3%) | F(37) = 23.3, p < 0.001, ICC = 0.92 |
| Play | Count | The subject engages in a behavioral pattern including gently biting, chasing, jumping and wrestling with the partner showing a relaxed body posture and facial expression | Pulled for Exploration + Reunion + Novel Object | [0] = 0 (67.7%)  [1,22] = 1 (32.3%) | F(37) = 19, p < 0.001, ICC = 0.90 |
| Greeting | Duration | The dog interacts in a friendly and relaxed manner with the partner, holding the ears back, visibly wagging the tail and/or licking the other one´s muzzle (the latest only in intraspecific tests) | Reunion | [0] = 0 sec (60.6%)  [0.1,20.0] sec = 1 (39.4%) | F(37) = 12.4, p < 0.001, ICC = 0.84 |
| Fear-related behaviors^1^ | Count | The dog shows a crouched body position, tail tucked between the legs or jumps away from the object | Pulled for Novel Object + Social Threat | [0] = 0 (36.4%)  [1,8] = 1 (63.6%) | F(37) = 6.29, p < 0.001, ICC = 0.73 |
| Synchronized behaviors | Count | The dog moves in the same direction as the partner within 2 seconds and at a distance closer than 2 meters (i.e. active locomotion, sniffing on the ground, barking) | Pulled for Exploration + Reunion + Novel Object | [0] = 0 (21.2%)  [1,3] = 1 (41.4%)  [4,20] = 2 (37.4%) | F(37) = 11.9, p < 0.001, ICC = 0.85 |
| Stress-related behaviors^2^ | Count | The dog shows yawning, body shaking, self-grooming, lips or nose licking, scratching | Pulled for Exploration + Reunion + Novel Object + Social Threat | [0,3] = 0 (39.4%)  [4,7] = 1 (31.3%)  [8,34] = 2 (29.3%) | F(37) = 71.5, p < 0.001, ICC = 0.97 |
| Marking | Count | The dog urinates, defecates, scent rolls or scratches the ground | Pulled for Exploration + Reunion + Novel Object | [0] = 0 (15.2%)  [1,2] = 1 (39.4%)  [3,16] = 2 (45.4%) | F(37) = 71.2, p < 0.001, ICC = 0.97 |
| Passive | Presence/absence | The dog does not show any specific reaction | Social threat | [0] = 53.5 %  [1] = 46.5 % | F(37) = 5.31, p < 0.001, ICC = 0.64 |
| Aggression | Presence/absence | The dog growls, snarls or snaps at the masked person | Social threat | [0] = 70.7 %  [1] = 29.3 % | F(37) = 9.11, p < 0.001, ICC = 0.81 |
| Friendly | Presence/absence | The dog moves towards the masked person visibly wagging the tail positioned at/under the median of the body | Social threat | [0] = 69.7 %  [1] = 30.3 % | F(37) = 7.62, p < 0.001, ICC = 0.75 |
| Hide behind the partner | Presence/absence | The dog positions itself behind the partner | Social threat | [0] = 84.8 %  [1] = 15.2 % | F(37) = 12.0, p < 0.001, ICC = 0.85 |
| Retreat | Count | The dog moves backwards | Social threat | [0] = 45.5 %  [>1] = 54.5 % | F(37) = 5.39, p < 0.001, ICC = 0.65 |
| Alternation of gaze between the partner and the experimenter | Presence/absence | The dog´s head orientation is towards the partner followed/preceded within 1 s by a look at the masked person | Social threat | [0] = 54.5 %  [1] = 45.5% | F(37) = 5.18, p < 0.001, ICC = 0.68 |
| Owner* | Duration | The subject looks at the owner present outside the enclosure and/or stands/sits within 2 body lengths from the owner | Pulled for Exploration + Reunion + Novel Object | [0, 60] sec = 0 (16.67%)  [60, 119] sec = 1 (20.83%)  [120, 179] sec = 3 (25%)  [>180] sec = 4 (37.5%) | F(37) = 15.8, p < 0.001, ICC = 0.85 |
| ^1^Following Barrera et al. 2010  ^2^Following Beerda et al. 1999  *only coded during dog-dog tests  ICC: Intraclass Correlation Coefficient | | | | |  |

| **Table S2. Rotated component matrix restricted to three components (Exploration, Separation, Reunion and Novel object test)** | | | |
| --- | --- | --- | --- |
|  |  | Components |  |
| Variable | Reference | Affiliation | Stress |
| Alternation of gaze between the partner and the novel object | **0.83** | -1.11 | 0.31 |
| Gaze at the partner | **0.75** | 0.12 | -0.34 |
| Greeting | **0.55** | 0.02 | 0.17 |
| Fear-related behaviors | **0.48** | 0.23 | -0.02 |
| Affiliative behaviors | -0.01 | **0.80** | 0.03 |
| Synchronized behaviors | 0.01 | **0.78** | 0.01 |
| Play | 0.26 | **0.48** | -0.19 |
| Stress-related behaviors | 0.10 | -0.15 | **0.76** |
| Marking | -0.07 | 0.07 | **0.74** |
| Eigenvalue | 2.06 | 1.48 | 1.23 |
| Variance explained | 22.86 | 16.47 | 13.70 |
| Cronbach´s alpha | 0.61 | 0.40 | 0.36 |
| Behaviors loadings in each factor in boldface. | | | |

| **Table S3. Clusters of dogs tested with a conspecific partner** | | | | | |
| --- | --- | --- | --- | --- | --- |
| Cluster | N | Variable | Mean | s.d. | Range |
| 1 (Friend) | 22 | Reference | -0.66 | 0.62 | -1.79; 0.49 |
|  |  | Affiliation | 1.18 | 0.55 | 0.33; 2.06 |
|  |  | Stress | -0.38 | 0.96 | -2.08; 1.05 |
| 2 (Independent) | 14 | Reference | -0.73 | 0.80 | -1.66; 0.98 |
|  |  | Affiliation | -0.68 | 0.31 | -1.23; -0.16 |
|  |  | Stress | -1.10 | 0.63 | -2.10; -0.07 |
| 3 (Insecure) | 34 | Reference | -0.08 | 0.84 | -1.46; 1.36 |
|  |  | Affiliation | -0.19 | 0.76 | -1.44; 1.66 |
|  |  | Stress | 0.73 | 0.70 | -0.65; 1.92 |

| **Table S4. Clusters of dogs tested with the owner** | | | | | |
| --- | --- | --- | --- | --- | --- |
| Cluster | N | Variable | Mean | s.d. | Range |
| 1 (Tense) | 19 | Reference | 0.66 | 0.76 | -0.82; 1.87 |
|  |  | Affiliation | -0.88 | 0.56 | -1.74; 0.42 |
|  |  | Stress | 0.18 | 0.69 | -0.66; 1.50 |
| 2 (Close) | 10 | Reference | 1.48 | 0.32 | 0.66; 1.81 |
|  |  | Affiliation | 0.65 | 0.98 | -0.97; 1.87 |
|  |  | Stress | -0.45 | 0.93 | -1.91; 0.92 |

| **Table S5. Clusters and raw behavioral variables** | | | | | | | | | |
| --- | --- | --- | --- | --- | --- | --- | --- | --- | --- |
| Cluster | Mean ± s.e.m. |  |  |  |  |  |  |  |  |
|  | Alternation of gaze | Gaze to the partner | Greeting | Fear | Affiliative | Synchronization | Play | Stress | Marking |
| Tense dog-owner | 1.53 ± 0.16 | 1.47 ± 0.18 | 0.42 ± 0.11 | 0.63 ± 0.11 | 0.05 ± 0.05 | 0.52 ± 0.14 | 0.16 ± 0.09 | 0.95 ± 0.19 | 1.60 ± 0.11 |
| Close dog-owner | 1.80 ± 0.13 | 1.90 ± 0.10 | 1.00 ± 0.00 | 1.00 ± 0.00 | 0.70 ± 0.15 | 1.30 ± 0.30 | 0.70 ± 0.15 | 0.50 ± 0.22 | 1.00 ± 0.21 |
| Independent | 0.29 ± 0.13 | 0.64 ± 0.23 | 0.21 ± 0.11 | 0.50 ± 0.14 | 0.00 ± 0.00 | 0.86 ± 0.14 | 0.14 ± 0.10 | 0.36 ± 0.17 | 0.50 ± 0.17 |
| Insecure | 0.85 ± 0.13 | 0.65 ± 0.12 | 0.41 ± 0.09 | 0.59 ± 0.09 | 0.15 ± 0.06 | 1.21 ± 0.12 | 0.29 ± 0.08 | 1.53 ± 0.10 | 1.56 ± 0.10 |
| Friend | 0.23 ± 0.09 | 0.95 ± 0.15 | 0.18 ± 0.08 | 0.64 ± 0.10 | 0.82 ± 0.08 | 1.77 ± 0.09 | 0.45 ± 0.11 | 0.41 ± 0.14 | 1.27 ± 0.16 |

| **Table S6. LMMs and GLMMs schematic results** | | | | |
| --- | --- | --- | --- | --- |
| **Response variables** | **Predictors** | | | |
|  | **Cluster classification** | **Breed** | **Order** | **Owner** |
| **Reference** | Close DO > Friend, Insecure, Independent DD;  Tense DO > Friend, Insecure, Independent DD | *ns* | *ns* | *ns* |
| **Affiliation** | Close DO > Insecure, Independent DD;  Tense DO < Friend, Insecure DD | *ns* | *ns* | *ns* |
| **Stress** | Tense DO < Insecure DD;  Tense DO > Independent DD | BC > other breeds | *ns* | *ns* |
| **Aggression** | *ns* | *ns* | *ns* | NA |
| **Retreat** | Close DO > Tense DO | *ns* | ↓ | NA |
| **Passive** | *ns* | *ns* | *ns* | NA |
| **Friendly** | *ns* | BC < other breeds | *ns* | NA |
| **Alternation of gaze between the partner and the experimenter** | Tense DO < Insecure DD | *ns* | *ns* | NA |
| Abbreviations: DO = dog-owner relationship type; DD= dog-dog relationship type; ns = not significant, NA = not available | | | | |

**Supplementary Figures:**


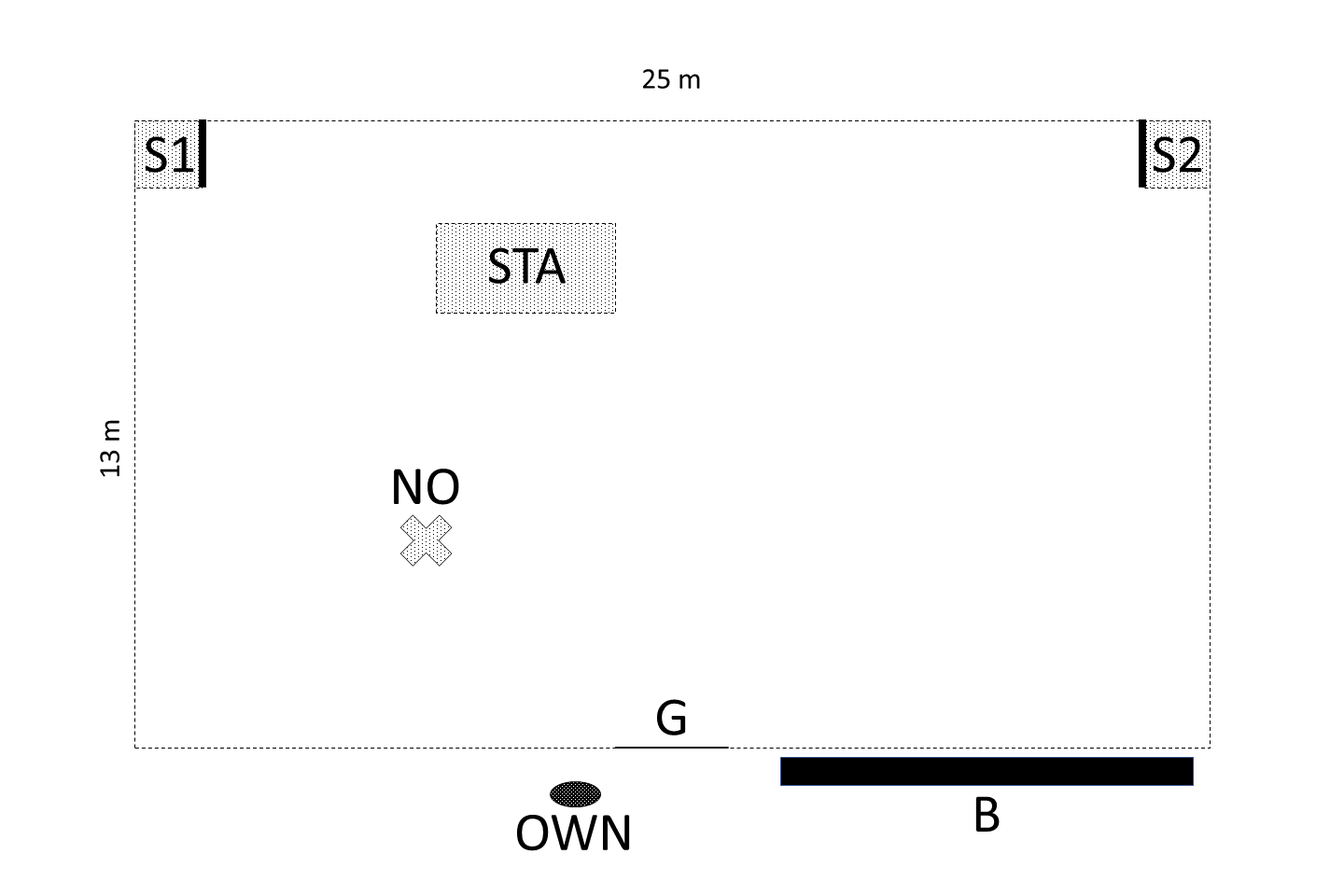


**Fig. F1:** Schematic representation of testing area A or B or C used in the present study. The two separation areas (S1 and S2, 1.5x1.5 m respectively) had an opaque fence only on one side, to allow visual separation of the two subjects while letting them see their owner (OWN, standing outside the enclosure) in the dog-dog tests. NO = position of the novel object used during the Novel Object test. STA = Social Threat area (2x4 m), where the dyads were enclosed when being approached by the masked experimenter. G = gate/entrance of the testing enclosure. B = visual barrier outside the testing enclosure. Subjects waited behind it (without witnessing) when the novel object was placed in the testing area before the Novel Object test. From behind the same barrier the masked experimenter appeared and started to walk towards the subjects during the Social Threat test.

**Fig. F2:** Schematic representation of the analyses conducted in the present study.

**Supplementary Methods:**

Dogs were tested either 1, 2 or 3 times: only once with a dog (N=10); only once with the owner (N=1); twice with 2 different dogs (N=9); twice, once with a dog, once with the owner (N=12); 3 times, twice with 2 different dogs, once with the owner (N=16). The only dog that was tested only once with the owner was supposed to participate in a second test with a conspecific partner, but for private reasons she could not take part in further tests. Still, data from this dog is also included.

**Supplementary Results:**

**R1: Principal Component Analysis: Exploration, Separation, Reunion, Novel Object**

Before running the PCA, we conducted some preliminary explorative analyses of the data that revealed that the same behavioral variable was correlated across subtests. For the variables "Gaze at the partner" and "Stress-related behaviors" we found that their amounts were correlated across subtests (Pearson’s or Spearman correlation depending on data distribution): Gaze at the partner: p<0.001 for all pairs of subtests; Stress-related behaviors: p<0.05 for all pairs of subtests except stress during separation and during reunion (p>0.05). For the other variables, the reason was that these behaviors (i.e. Affiliative behaviors, Play, Fear-related behaviors, Marking) occurred in very small numbers in all subtests. Therefore, we decided to sum them up.

With the PCA, we extracted three components based on their eigen value (> 1), including all the 9 behavioral variables coded during the Exploration, Separation, Reunion and Novel Object, which accounted for 53.03% of the total variance (Bartlett´s test: X2(36) = 102.71, p < 0.01, KMO = 0.56 see Table 2). The first component accounted for 22.86% of the variance and it included: Alternation of gaze between the partner and the novel object, Gaze at the partner, Greeting, Fear-related behaviors. We labeled this component “Reference” (Cronbach´s alpha = 0.60). The second component accounted for 16.47% of the total variance and it included: Affiliative behaviors, Synchronized behaviors and Play. We labeled it “Affiliation” (Cronbach´s alpha = 0.40). The third component, which we labeled “Stress” included Stress-related behaviors and Marking, accounting for 13.70% of the total variance (Cronbach´s alpha = 0.36; see Table 2).

**R2: Intra- vs. inter-specific relationships**:

The Discriminant Function Analysis on the dog-owner clusters correctly classified 84.8% of the cases (84.3% of dog-dog tests and 86.2% of dog-owner tests). Two-tailed binomial tests showed that these classifications were significantly higher than by chance (p < 0.001 for both groups), suggesting that dogs behaved differently when tested with their owners and with their conspecific partners. The analysis extracted one discriminant function which reliably discriminated between the clusters (X2(3) = 52.76, p < 0.001, Wilk´s lambda=0.58). Reference was the behavioral variable mostly contributing to this difference (canonical discriminant function coefficients: Reference = 0.98, Affiliation = - 0.45, Stress = - 0.05). The Box M multivariate test for equality of variable covariance revealed inequality between the clusters (Box M test: 6.34, F = 1.01, p = 0.42).

**R3: Partner species as a possible source of variation in the subjects’ behaviors**

In order to investigate whether the dog partners (having presumably less controlled behavior than the owners) could cause a higher variation in the subjects' behavior than the owners as partners, we conducted Bartlett’s tests on the raw behavioral variables, comparing dogs tested with a dog partner and with the owner. All tests resulted non-significant (all p > 0.05), failing to reject the null hypothesis that the two groups were characterized by homogeneous variance (Fear-related behaviors: K2(1) = 0.02, df = 1, p > 0.05; play: K2(1) = 0.03, p > 0.05; Synchronized behaviors: K2(1) = 0.34, p > 0.05; Affiliative behaviors: K2(1) = 1.26, p > 0.05; Gate at the partner: K2(1) = 0.37, p > 0.05; Stress-related behaviors: K2(1) = 1.01, p > 0.05; Marking: K2(1) = 3.77, p > 0.05; Greeting: K2(1) = 0.18, p > 0.05; Alternation of Gaze between the partner and the Novel Object: K2(1) = 0.25, p > 0.05).

**R4: Possible influence of the owner present outside the enclosure during dog-dog tests**

In order to check whether the presence of the owner distracted the dogs tested in the intraspecific condition, we coded the time spent looking at the owner present outside the enclosure and in proximity (within 2 body lengths) to the owner in the dog-dog tests (variable “Owner”). We then included this variable in the final models as main effect. The variable did not have a significant effect on all models we ran: “Reference” (estimate ± s.e.m. = 0.06 ± 0.08, t = 0.76, p > 0.05), “Affiliation” (estimate ± s.e.m. = - 0.02 ± 0.07, t = - 0.35, p > 0.05), and “Stress” (estimate ± s.e.m. = - 0.13 ± 0.08, t = - 1.70, p > 0.05).

**R5: Other factors possibly influencing subjects’ behaviors: order of testing, breed**

Since some dogs were tested up to three times, we analyzed if order of testing influenced how dogs behaved during the study by including “Order” in the linear mixed models (ordinal variable ranging from 1 to 3), but none of the variables was significantly affected by order of testing: “Reference” (estimate ± s.e.m. = - 0.07 ± 0.12, t = - 0.61, p > 0.05), “Affiliation” (estimate ± SE= -0.09 ± 0.11, t = - 0.88, p > 0.05), “Stress” (estimate ± s.e.m. = 0.16 ± 0.11, t = -1.47, p > 0.05), “Aggression” (estimate ± s.e.m. = - 0.22 ± 1.07, t = - 0.21, p > 0.05), “Friendly” (estimate ± s.e.m. = 1.32 ± 1.26, t = 1.05, p > 0.05), “Alternation of gaze between the experimenter and the partner” (estimate ± s.e.m. = - 1.36 ± 1.38, t = - 0.99, p > 0.05), and “Passive” (estimate ± s.e.m. = 0.14 ± 0.15, t = 0.99, p > 0.05). However, we found that more dogs were exposed to the Social Threat test, less they retreated (estimate ± s.e.m. = - 0.20 ± 0.08, t = - 2.63, p = 0.01). Still, we can conclude that having been tested more than once did not lead to habituation or sensitization to the testing procedure, besides in the case of retreating during the Social Threat test.

Moreover, we included the breed of the dog (Border Collie vs. other breeds) in the linear mixed models as predictor. We found that Border Collie dogs did not behave differently from the other dogs regarding the variable “Reference” (Border Collie vs. other breeds: estimate ± s.e.m. = 0.13 ± 0.20, t = 0.65, p > 0.05), “Affiliation” (Border Collie vs. other breeds: estimate ± s.e.m. = 0.28 ± 0.16, t = 1.80, p > 0.05), “Aggression” (Border Collie vs. other breeds: estimate ± s.e.m. = - 2.89 ± 2.68, t = 1.08, p > 0.05), “Retreat” (Border Collie vs. other breeds: estimate ± s.e.m. = - 0.08 ± 0.17, t = - 0.48, p > 0.05), “Passive” (Border Collie vs. other breeds: estimate ± s.e.m. = 0.08 ± 0.25, t = 0.32, p > 0.05), and “Alternation of gaze between the experimenter and the partner” (Border Collie vs. other breeds: estimate ± s.e.m. = - 2.74 ± 2.08, t = - 1.31, p > 0.05), but we found that Border Collie dogs scored higher in the variable “Stress” than the other dogs (Border Collie vs. other breeds: estimate ± s.e.m. = 0.48 ± 0.20, t = 2.39, p = 0.02) and that they were less likely to show a friendly reaction than dogs belonging to other breeds (Border Collie vs. other breeds: estimate ± s.e.m. = - 8.02 ± 1.90, t = - 4.22, p < 0.001).

**R6: Behavioral styles: validation of cluster classification**

DFA on the dog-dog clusters correctly classified 98.60% of the cases (100% of Cluster 1, 92.9% of Cluster 2 and 100% of Cluster 3). Two-tailed binomial tests showed that the correct classifications were significantly higher than by chance for all groups (p < 0.001 for all clusters), validating the cluster classification obtained with the cluster analysis. The analysis extracted two discriminant functions, which reliably discriminated between the clusters (first function: X2 (6) = 128.00, p < 0.001, Wilk´s lambda = 0.14; second function: X2(2) = 42.70, p < 0.001, Wilk´s lambda = 0.51). The first function´s canonical coefficients revealed that Affiliation mostly contributed to the categorization of the clusters (canonical discriminant function 1 coefficients: Reference = 0.76, Affiliation = - 0.99, Stress = 0.65). The second function´s canonical coefficients, however, revealed that Stress was the most contributing variable (canonical discriminant function 2 coefficients: Reference = 0.18, Affiliation = 0.54, Stress = 0.76). The Box M multivariate test for equality of variable covariance revealed inequality between the clusters (Box M test: 5.11, F = 0.81, p = 0.56).

The DFA on the dog-owner clusters correctly classified 100% of the cases (100% of Cluster 1 and 100% of Cluster 2). Two-tailed binomial tests showed that these classifications were significantly higher than by chance (p < 0.001 for both groups), validating the cluster classification obtained with the cluster analysis. The analysis extracted one discriminant function which reliably discriminated between the clusters (X2(3) = 32.87, p < 0.001, Wilk´s lambda = 0.28). Affiliation was the behavioral variable mostly contributing to this difference (canonical discriminant function coefficients: Reference = 0.54, Affiliation = 1.00, Stress = - 0.61). The Box M multivariate test for equality of variable covariance revealed inequality between the clusters (Box M test: 0.09, F = 0.08, p = 0.77).

See Table S5 for more information on how dogs present in the different clusters differed for the raw behavioral variables included in the PCA.

**R7: Factors possibly affecting cluster membership: household, individual, sex, breed**

In general, different dogs from the same household did not always show the same behavioral pattern towards the same owner: in fact, in 5 households all dogs fell in the same dog-owner cluster, while in 6 households, different dogs fell in different dog-owner clusters, suggesting that the cluster classification did not depend on the household membership (e.g. on the owner´s characteristics). Similarly, only in one case all dogs belonging to the same household fell in the same dog-dog cluster, while in the other 13 cases dogs belonging to the same households fell in different dog-dog clusters, suggesting that the behavioral patterns dogs showed with conspecifics were not determined by their household.

Cluster classification did not depend on the sex composition of the pair either: in fact, the distribution of same-sex and mixed-sex dyads did not differ across clusters (Chi square test: dog-dog clusters: X2(4) = 2.88, p = 0.24; dog-owner clusters: X2(1) = 0.82, p = 0.37).

Moreover, belonging to a Border Collie breed (in contrast of being a mixed-breed dog or belonging to another breed) did not influence cluster classification (Chi-square test: X2(4) = 5.51, p = 0.24).
